# Development of a *Rhizoctonia solani* AG1-IB Specific Gene Model Enables Comparative Genome Analyses between Phytopathogenic *R*. *solani* AG1-IA, AG1-IB, AG3 and AG8 Isolates

**DOI:** 10.1371/journal.pone.0144769

**Published:** 2015-12-21

**Authors:** Daniel Wibberg, Oliver Rupp, Jochen Blom, Lukas Jelonek, Magdalena Kröber, Bart Verwaaijen, Alexander Goesmann, Stefan Albaum, Rita Grosch, Alfred Pühler, Andreas Schlüter

**Affiliations:** 1 Institute for Genome Research and Systems Biology, CeBiTec, Bielefeld University, Bielefeld, Germany; 2 Bioinformatics and Systems Biology, Gießen University, Gießen, Germany; 3 Leibniz-Institute of Vegetables and Ornamental Crops, Großbeeren, Germany; The University of Wisconsin - Madison, UNITED STATES

## Abstract

*Rhizoctonia solani*, a soil-born plant pathogenic basidiomycetous fungus, affects various economically important agricultural and horticultural crops. The draft genome sequence for the *R*. *solani* AG1-IB isolate 7/3/14 as well as a corresponding transcriptome dataset (Expressed Sequence Tags—ESTs) were established previously. Development of a specific *R*. *solani* AG1-IB gene model based on GMAP transcript mapping within the eukaryotic gene prediction platform AUGUSTUS allowed detection of new genes and provided insights into the gene structure of this fungus. In total, 12,616 genes were recognized in the genome of the AG1-IB isolate. Analysis of predicted genes by means of different bioinformatics tools revealed new genes whose products potentially are involved in degradation of plant cell wall components, melanin formation and synthesis of secondary metabolites. Comparative genome analyses between members of different *R*. *solani* anastomosis groups, namely AG1-IA, AG3 and AG8 and the newly annotated *R*. *solani* AG1-IB genome were performed within the comparative genomics platform EDGAR. It appeared that only 21 to 28% of all genes encoded in the draft genomes of the different strains were identified as core genes. Based on Average Nucleotide Identity (ANI) and Average Amino-acid Identity (AAI) analyses, considerable sequence differences between isolates representing different anastomosis groups were identified. However, *R*. *solani* isolates form a distinct cluster in relation to other fungi of the phylum *Basidiomycota*. The isolate representing AG1-IB encodes significant more genes featuring predictable functions in secondary metabolite production compared to other completely sequenced *R*. *solani* strains. The newly established *R*. *solani* AG1-IB 7/3/14 gene layout now provides a reliable basis for post-genomics studies.

## Introduction

The basidiomycetous fungus *Rhizoctonia solani* Kühn (teleomorph *Thanatephorus cucumeris* [Frank] Donk) is a worldwide prevalent soil-borne plant pathogen. It causes diseases on many economically important crops including sugar beet, potato, wheat, rape, maize, soybean, rice, cabbage, cauliflower, tomato and lettuce as well as ornamental plants and forest trees [1]. Currently, *R*. *solani* isolates are classified into 13 distinct groups named anastomosis groups (AGs) based on occurrence of hyphal interaction between isolates of the same AG [2].

The genomic era for *R*. *solani* AG1 has been initiated by publication of the draft genome sequences for the AG1-IB isolate 7/3/14 [3,4] and the AG1-IA isolate B275 [5]. The genome sequences for AG3 and AG8 isolates became available quite recently [6,7]. The *R*. *solani* AG1-IB genome project provided first insights into the genome size and structure, gene content and functional annotation of the sequenced isolate. Moreover, the isolate’s diploid nature was followed at the genomic level. However, gene prediction for *R*. *solani* AG1-IB at that time was based on a gene model developed for the related fungus *Coprinopsis cinerea* of the order *Agaricales*, whereas *R*. *solani* belongs to the order *Cantharellales*. Recently, transcriptome sequence data obtained by high-throughput (HT) sequencing of Expressed Sequence Tags (ESTs) became available for *R*. *solani* AG1-IB [8]. *R*. *solani* ESTs provided information on genes transcribed under the conditions tested and their exon structure. Moreover, putative pathogenicity determinants were recognized within the EST dataset.

In general, gene prediction in fungal genomes is limited by the accuracy of gene prediction programs. *Ab initio* gene prediction for eukaryotes is based on methods that exploit structural and statistical properties of coding sequences that differ for each organism. Gene prediction rules are compiled from correctly recognized coding sequences of the organism of interest or one of its close relatives. Different gene prediction tools for eukaryotes have been developed over the last 30 years, e.g. GeneMark [9], GenomeScan [10], AUGUSTUS [11] and others. These tools are routinely applied for automatic gene finding in eukaryotic genomes. However, the performance of existing gene prediction tools still is unsatisfactory [11]. Recently, improvements in fungal gene prediction were achieved by using RNA-Seq and homology information implemented in the SnowyOwl pipeline [12]. Another important factor affecting gene model development for gene prediction is the degree of fragmentation in eukaryotic genome projects after assemblies. Frequently genes are split between contigs. However, reliable gene prediction algorithms and models are needed to enable comparative gene-based analyses between the genomes of related species and sub-species. Bioinformatics tools, such as the comparative genomics platform EDGAR [13], address the identification of orthologous genes in different genomes and the calculation of the core genome and unique genes for each genome within a set of genomes to be compared. Commonly, comparative genome analyses were undertaken to estimate unique genome features of an isolate of interest and to determine its relationship to related and reference strains. First comparative analyses of two *R*. *solani* isolates, namely those representing AG1-IA and AG1-IB, were recently published [14]. However, gene-based comparative analyses for all sequenced *R*. *solani* isolates representing different AGs have not been undertaken so far.

The objective of this study was to take advantage of the *R*. *solani* AG1-IB Expressed Sequence Tag dataset [8] as a valuable resource for improving recognition and prediction of genes in the genome of this isolate. In particular, an *R*. *solani* AG1-IB specific gene model was developed and applied to enable comparative genome analyses comprising other *R*. *solani* genome sequences, namely those of *R*. *solani* isolates classified as belonging to AG1-IA, AG1-IB, AG3 and AG8 [3–7]. Since the *R*. *solani* AGs included in this comparison differ in host specificity regarding their pathogenic interactions, it was hypothesized that unique genes within their genomes should reflect specific characteristics of the corresponding isolate. It was attempted to identify candidate genetic determinants that may play a role in host-specific pathogenicity, especially for *R*. *solani* AG1-IB. Moreover, application of the newly developed *R*. *solani* gene model was expected to enable recognition of new, so far missed genes in the *R*. *solani* AG1-IB genome and hence improvements regarding gene content and functional gene annotation were in the focus of this study.

## Material and Methods

### Sequence datasets for the development of an R. solani AG1-IB specific gene model

The improved genome sequence [EMBL: CDGK01000001 –CDGK01018395 (Contigs); LN679100 –LN679996 (Scaffolds)] [4] and the expressed sequence tag (EST) dataset [EMBL:HG330226-HG379789] [8] for *R*. *solani* AG1-IB (isolate 7/3/14) were used for the development of an AG1-IB specific gene model. Both datasets were established by applying the gsAssembler software (2.6/2.8).

### Mapping of ESTs onto the *R*. *solani* AG1-IB 7/3/14 genome for generation of a gene structure file

The combined *R*. *solani* AG1-IB 7/3/14 EST dataset [8] was mapped onto the *R*. *solani* AG1-IB 7/3/14 genomic contigs as described previously using the mapping program GMAP [15] to identify gene encoding regions. For classification of alternative splicing events, the software tool ASTALAVISTA was used [16]. Finally, the manually curated output of GMAP, a gene structure file (.gff), was used as training set for the development of an *R*. *solani* AG1-IB 7/3/14 gene model.

### Gene model training, gene prediction based on the training dataset and evaluation of different gene models

The gene structure file computed by means of GMAP was imported into the AUGUSTUS training system to deduce a parameter dataset for *R*. *solani* AG1-IB. A parameter dataset comprises Markov chain transition probabilities of coding (exon) and non-coding (intron or intergenic) regions. For each species, there are also 'meta parameters' such as the order of the Markov chain, or the size of the window used for the splice-site models. The parameter dataset includes species specific information such as intron and exon length distributions, splice-site patterns, translation start-site patterns or branch point regions of introns.

Gene prediction was accomplished by applying AUGUSTUS version 3.0.3 [11] on *R*. *solani* AG1-IB contigs by applying the newly developed gene model for this species. To evaluate gene prediction results, identified genes were compared to EST sequences by means of BLASTn [17], to unassembled transcriptome reads by means of bowtie2 [18] and to references gene products deposited in the NCBI database by means of BLASTp [17] In addition, the gene prediction results were compared to the gene prediction based on the *C*. *cinerea* gene model. To verify intron-exon and exon-intron borders, the tool Geneious version 6.0.3 created by Biomatters (http://www.geneious.com/) was used for multiple alignments applying default settings. Transcript isotig mappings on *R solani* AG1-IB contigs were taken as references and alignment results were compared to the gene structure obtained by gene prediction. The *R*. *solani* AG1-IB gene prediction based on the specific parameter set is publicly available in the next version of AUGUSTUS and on the website http://bioinf.uni-greifswald.de/webaugustus/prediction/create. The newly predicted genes were annotated by means of the automatic annotation pipeline in SAMS [19,20] and a modified GenDB 2.0 version [20,21]. Mobile genetic elements belonging to the Long Terminal Repeat (LTR) group were identified and annotated by applying thirteen Hidden-Markov-Model (HMM) profiles of eight different protein domains (INT [PF14657, PF12835, PF00665, PF02920], RT [PF00078], AP [PF00847], RNase H [PF00075], Gag [PF00540, PF00607, PF08705, PF02093, PF02337, PF01141, PF01140, PF02228, PF03732, PF08723], Chromo [PF00385], RVT_thumb [PF06817], RVT_connect [PF06815]) by applying the tools LTRharvest [22] and LTRdigest [23].

### Comparative genome analyses for *R*. *solani* isolates representing different anastomosis groups

Annotated genome information for the *R*. *solani* AG1-IA isolate B275 [GenBank: AFRT00000000], *R*. *solani* AG8 isolate WAC10335 [GenBank: AVOZ0000000] and *R*. *solani* AG3 isolate Rhs1AP [GenBank: JATN0100000] are publicly available. The genomes of these *R*. *solani* isolates were used for comparative genome analyses. Comparative analyses between the *R*. *solani* AG1-IB 7/3/14 draft genome and the *R*. *solani* genomes listed above were accomplished using a modified version of the comparative genomics program EDGAR designed to handle eukaryotic genomes and their multi-exon genes [13]. Comparative analyses comprised identification of orthologous genes and classification of genes as core genes or singletons.

### Phylogenetic analysis of basidiomycetous fungi based on core genes

Phylogenetic relationships for *R*. *solani* AG1-IB 7/3/14, *R*. *solani* AG1-IA, *R*. *solani* AG3 and *R*. *solani* AG8 as well as the related fungi *Coprinopsis cinerea* okayama 7#130 [24], *Piriformospora indica* DSM 11827 [25] and *Cryptococcus neoformans* var. neoformans JEC21 [26] were computed by means of EDGAR [13]. The core genome of all selected fungi was calculated within EDGAR and based on all core genes, phylogenetic distances were calculated from multiple sequence alignments. Phylogenetic trees were constructed from concatenated core gene alignments using PHYLIP [27] as previously outlined in detail [13]. In addition, average nucleotide identity (ANI) and average amino acid identity analyses (AAI) were performed as described previously [28,29] to determine the relationship between *R*. *solani* isolates representing different anastomosis groups (AGs). For determination of thresholds regarding fungal species, ANI and AAI analyses were performed comprising the genomes of *Aspergillus niger* SH-2 (AUZU01), *Asperigillus niger* ATCC1015 (ACJE01), *Candida albicans* WO-1 (AAF001), *Candida albicans* A20 (AVAX01), *Metarhizium anisopliae* BRIP 53293 (APNC01), *Metarhizium anisopliae* BRIP 53284 (APNB01), *Crytococcus neoformans* var. neoformans JEC21 (AEO17341-56), *Crytococcus neoformans* var. neoformans B-3501A (CM000040-53), *Fusarium oxysporum* f.sp. cubense race 1 (AMGP01) and *Fusarium oxysporum* f.sp. cubense race 4 (AMGQ01).

## Results and Discussion

### Development of an *R*. *solani* AG1-IB gene model exploiting EST mapping results

The *R*. *solani* AG1-IB 7/3/14 draft genome sequence was established recently [3,4]. Likewise, Expressed Sequence Tags (ESTs) were deeply sequenced for this *R*. *solani* isolate grown in different media [8]. These sequence datasets (genomic and EST sequences) were now used to deduce an *R*. *solani* specific gene model which then was applied to uncover new *R*. *solani* AG1-IB genes that were missed in previous gene predictions on genomic contigs or are not represented in the EST datasets. Accordingly, isotigs (transcript isoforms) from the *R*. *solani* AG1-IB 7/3/14 EST datasets were mapped onto the improved corresponding genome sequence to define constraints for the prediction of exon-intron and intron-exon junctions and to determine coding-region start-sites [8]. An *R*. *solani* gene model was computed by applying the eukaryotic gene prediction program AUGUSTUS [11] that previously was used in many fungal genome annotation projects, e.g. for the two *Basidiomycota* species *L*. *bicolor* [30] and *C*. *cinerea* [24]. Gene prediction in *R*. *solani* AG1-IB based on a specific gene model is the prerequisite for reasonable comparative analyses between the genomes of *R*. *solani* isolates representing different AGs.

Exact exon-intron junctions were deduced by mapping of isotig sequences from the EST datasets onto genomic sequences by means of the mapping tool GMAP. In total, 13,185 of 20,202 *R*. *solani* AG1-IB 7/3/14 isotigs were mapped onto the genome with more than 95% sequence identity and more than 90% template coverage. In most cases, only one isotig of a specific isogroup was mapped when strict mapping settings were applied. The following aspects may explain why 35% of the isotigs were not mapped on the *R*. *solani* genome: i) The genome assembly still is fragmented causing disruption of genes that are split between contigs. ii) Normalization in the course of library preparation for EST sequencing may have led to the enrichment of isotigs originating from rare nuclei that are not completely represented in the genome assembly. According to academic opinion, rare nuclei may be present due to the heterokaryotic nature of *R*. *solani* AG1-IB. However, the high degree of consistency between the genome assembly and the *de novo* transcriptome assembly revealed that the gsAssembler (version 2.6/2.8) is an appropriate tool for genome as well as transcriptome assemblies.

**Fig 1:**
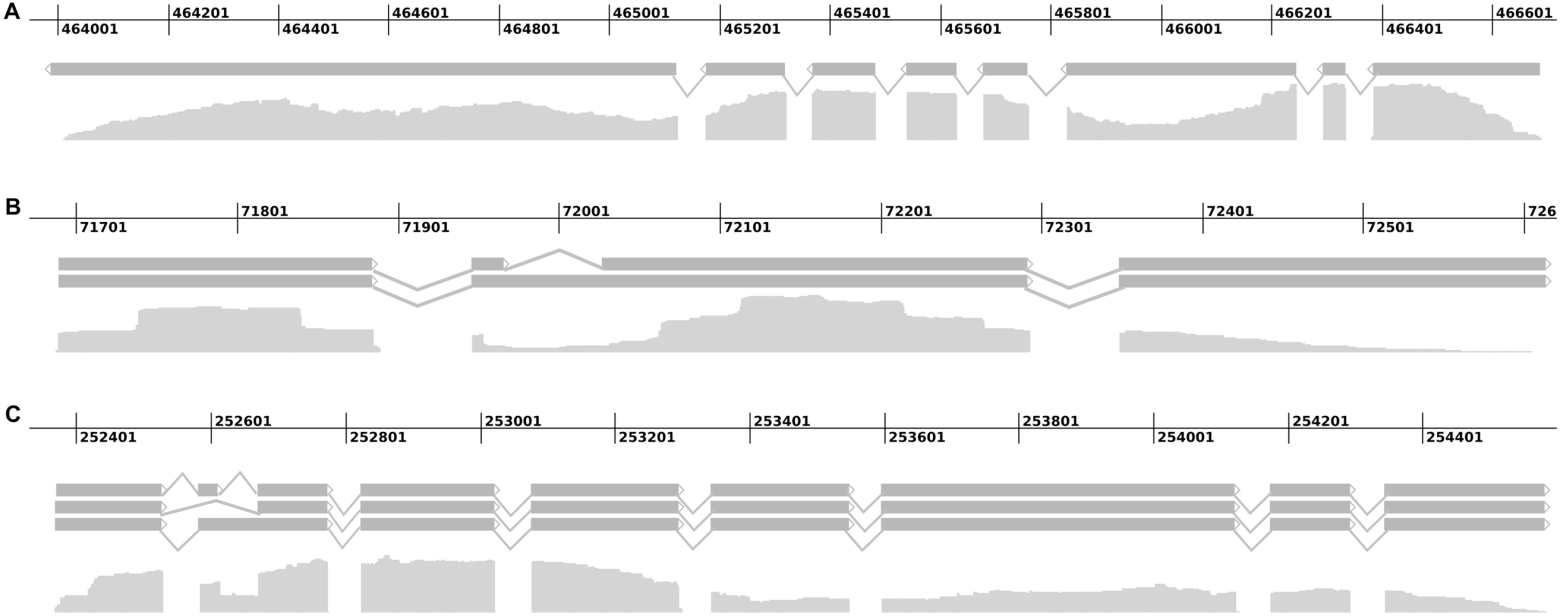
Alternative transcript splicing in *R*. *solani* AG1-IB 7/3/14. Transcript isoforms for selected genes are shown. Each line with bars represents a transcript isoform. The pileup track below the isoforms shows the amount of reads that were mapped to the corresponding genomic region. (A) A gene for which only one transcript isoform was identified. Sharp intron-exon borders are visible. (B) Intron retention is the most common alternative splicing event observed in *R*. *solani* AG1-IB. (C) A gene whose transcript exists in three isoforms featuring exon-skipping as well as intron-retention.

Visual inspection of aligned transcripts revealed alternatively spliced transcripts. Further analyses applying ASTALAVISTA [16], a bioinformatics tool for the analysis of alternative splicing events, uncovered that 4,796 alternative splicing events are represented by the mapped transcripts including 2,780 'intron-retention' events, 239 'alternative acceptor-site' events, 102 'alternative donor-site' events, 12 'exon-skipping' events and 1663 events that could not be classified into one of the aforementioned categories (Fig 1). These observations are in accordance with the findings of McGuire *et al*. [31] who showed that 'intron-retention' is the most prevalent alternative splicing event in fungi and that alternative acceptor-sites occur more frequently than alternative donor-sites. Transcriptome mapping results were retained in a file which then was applied as training file within AUGUSTUS for gene prediction on the *R*. *solani* AG1-IB 7/3/14 genome. This approach led to computation of an *R*. *solani* AG1-IB specific parameter set defining constraints such as translation initiation start sites, translation end points, acceptor (3’) splice sites, donor (5’) splice sites, exon and intron regions [32] which then were applied for gene prediction in this species.

### The newly developed gene model improves gene prediction in the *R*. *solani* AG1-IB genome

Gene prediction results on *R*. *solani* AG1-IB genomic contigs applying the new *R*. *solani* AG1-IB specific parameter set were compared to those obtained with the *C*. *cinerea* parameter set as described previously [3]. In general, gene predictions based on the two different parameter sets featured different values in all categories (Table 1). For instance, 12,616 genes were predicted by applying the new model, whereas only 11,157 genes were recognized in the approach using the *C*. *cinerea* gene model.

**Table 1:** Comparison of gene prediction results applying the specific *vs*. the *C*. *cinerea* gene model on the *R*. *solani* AG1-IB 7/3/14 draft genome.

Features	Specific gene model (based on *R*. *solani* AG1-IB gene structure file^1^)	Previous gene model (*C*. *cinerea* model^2^)
**Number of predicted genes**	12,616	11,157
**Average gene length**	1788 bp	1541 bp
**Average number of exons per gene**	6.26	6.41
**Average exon length**	218.71 bp	190.45 bp
**Average intron length**	78.12 bp	68.17 bp
**CDSs matching an EST of the transcriptome dataset (%** ^3^ **)**	9,595 (94.99%)	8,702 (86.74%)
**CDSs not matching an EST in the transcriptome dataset**	3,021	2,455
**CDSs having the same start and stop-position as predicted for the corresponding reference EST**	6,136	3,643

^**1**^ Gene model developed in this work based on GMAP mapping of *R*. *solani* AG1-IB Expressed Sequence Tags (ESTs)

^**2**^ Gene model applied in previous gene predictions for the *R*. *solani* AG1-IB genome [3]

^**3**^ 10,101 ESTs representing different isogroups (as described in chapter 3.1.) correspond to 100%

To identify the most appropriate gene prediction approach, gene products deduced from predicted genes were analyzed with the program BLASTp in comparison to those deduced from EST isogroups by translation. In total, 9,595 of 10,101 gene products from the latter approach were identified in the gene prediction based on the new gene model (94.99%), whereas only 86% of the gene products were identified using the *C*. *cinerea* gene model (Table 1). More than 3000 genes that are not represented within the EST dataset were predicted by application of the *R*. *solani* AG1-IB specific gene model. A total of 1256 of these so far unrecognized genes could be corroborated by mapping of non-assembled transcriptome reads onto them. For the remaining 1765 new genes, 1289 homologous references genes were detected in the NCBI database. These mainly originate from *R*. *solani* AG3 and hence represent orthologous genes in both isolates. Only 476 new *R*. *solani* AG1-IB genes could neither be supported by EST-mapping nor by database homologs.

Based on Pfam annotation, most of the newly identified genes encode hypothetical proteins (~1400). However, also genes encoding cytochromes P450 and enzymes potentially involved in degradation of plant cell wall components were predicted (see S1 Table).

**Fig 2:**
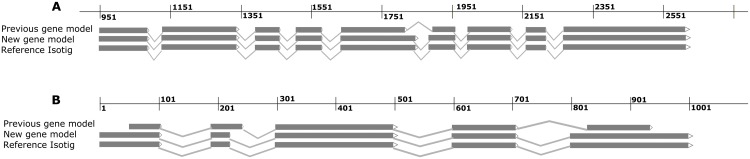
Comparison of gene prediction results applying the *R*. *solani* AG1-IB specific and the *C*. *cinerea* gene model. The diagrams exemplarily illustrates the gene prediction results obtained by application of the new gene model based on *R*. *solani* AG1-IB gene structure data and the previous gene model based on *C*. *cinerea* parameters. (A) Contig84 and (B) Contig25832 were chosen as examples for gene prediction. Corresponding EST sequence were mapped to the contigs for reference purposes. Based on isotig mapping, wrongly detected intron-exon junctions could be detected in case of the gene prediction by the *C*. *cinerea* model. The black line above the alignment results represents the scale in base pairs [bp].

EST mappings also verify rules for exon-intron and intron-exon junctions as defined by the *R*. *solani* AG1-IB specific gene model. Gene predictions based on the two different gene models exemplarily are shown in Fig 2 for a genomic region represented by a selected contig. Application of the new gene model led to the identification of 5735 genes that were missed or insufficiently recognized in the previous approach. These genes were automatically annotated within the Sequence Analysis and Management System SAMS [19] as well as a modified GenDB 2.0 version [21]. These systems assigned annotations and features with high confidence values to 3,514 of 5,735 new *R*. *solani* AG1-IB 7/3/14 genes. However, most of the newly predicted genes received annotations such as 'hypothetical protein' or 'uncharacterised protein' illustrating insufficient functional characterization of fungal genomes from members of the genus *Rhizoctonia* in databases. SAMS and GenDB annotation pipelines assigned 185 gene names, 588 EC numbers and 1050 KOG numbers to the new *R*. *solani* AG1-IB 7/3/14 genes (S1 Table).

Most of the functionally annotated and newly identified genes belong to retrotransposons or retroviruses. Transposable elements with long terminal direct repeats (LTR TEs) are one of the extensively studied group of mobile genetic elements [33]. In total, 231 LTR retrotransposons were identified in the *R*. *solani* AG1-IB 7/3/14 genome by means of the LTRharvest tool [22]. Among these elements, 129 LTR retrotransposons represent the Ty3/Gypsy and 66 the Ty1/Copia type. Likewise, other members of the phylum *Basidiomycota* such as *Postia placenta*, *Laccaria bicolor*, *Coprinopsis cinerea* and *Phanerochaete chrysosporium* also mainly harbor Ty3/Gypsy LTR retrotransposons [33].

New genes encoding enzymes predicted to be involved in degradation of cellulosic material, lignocellulose and cutin, melanin formation and synthesis of other secondary metabolites were identified [8,34]. Moreover, genes for different cytochromes P450, non-ribosomal peptide-synthetases, a phenol reductase and enzymes involved in alkaloid synthesis potentially extend the functional context of toxin synthesis in *R*. *solani* AG1-IB.

To summarize, application of the *R*. *solani* AG1-IB specific gene model revealed 5735 so far non-recognized genes, some of which may have a function in the context of fungus-plant interaction.

### Comparative genome analyses for different *R*. *solani* isolates revealed remarkable differences between members representing anastomosis groups AG1, AG3 and AG8

Four *R*. *solani* draft genome sequences representing AG1-IA [5], AG1-IB [3,4], AG3 [6] and AG8 [7] are available to date. A comparative analysis regarding genome features for these *R*. *solani* draft genomes is shown in Table 2. *R*. *solani* AG1-IA possesses the smallest draft genome that approximately is six million bases smaller than the *R*. *solani* AG1-IB 7/3/14 genome and only comprises two-thirds of the *R*. *solani* AG1-IB 7/3/14 gene content. The *R*. *solani* AG1-IB 7/3/14 mitochondrial (mt) genome also is 15 kb larger than the AG1-IA mt-genome and encodes seven additional genes. The genome of *R*. *solani* AG8 is slightly larger than that of isolate AG1-IA and has the smallest mt-genome of all sequenced *R*. *solani* strains. However, it has the highest coding density and the largest number of predicted genes within the set of sequenced *R*. *solani* isolates. *R*. *solani* AG3 has the largest draft genome size, as well as the largest mitochondrial genome with a size of approximately 236 kb [35].

**Table 2:** Genome features of completely sequenced *R*. *solani* isolates.

Features	*R*. *solani* AG1-IA ^1^	*R*. *solani* AG1-IB ^2^	*R*. *solani* AG3 ^3^	*R*. *solani AG8* ^4^
**No. of scaffolds**	2,649	879	328	857
**Scaffold length**	37.09 Mb	42.80 Mb	51.71 Mb	39.82 Mb
**No. of CDSs**	10,489	12,713	12,726	13,952
**GC-content**	47.61%	48.10%	48.40%	48.80%
**Size of mitochondrial genome**	147,264 bp	162,751 bp	235,849 bp	139,993 bp
**CDSs in mitochondrial genome**	21	28	139	53
**GC content of mitochondrial genome**	33.93%	36.41%	35.91%	35.32%
**tRNAs within mitochondrial genome**	26	25	26	17

^1^ [5]

^2^ [3,4]

^3^ [6,35]

^4^ [7]

**Fig 3:**
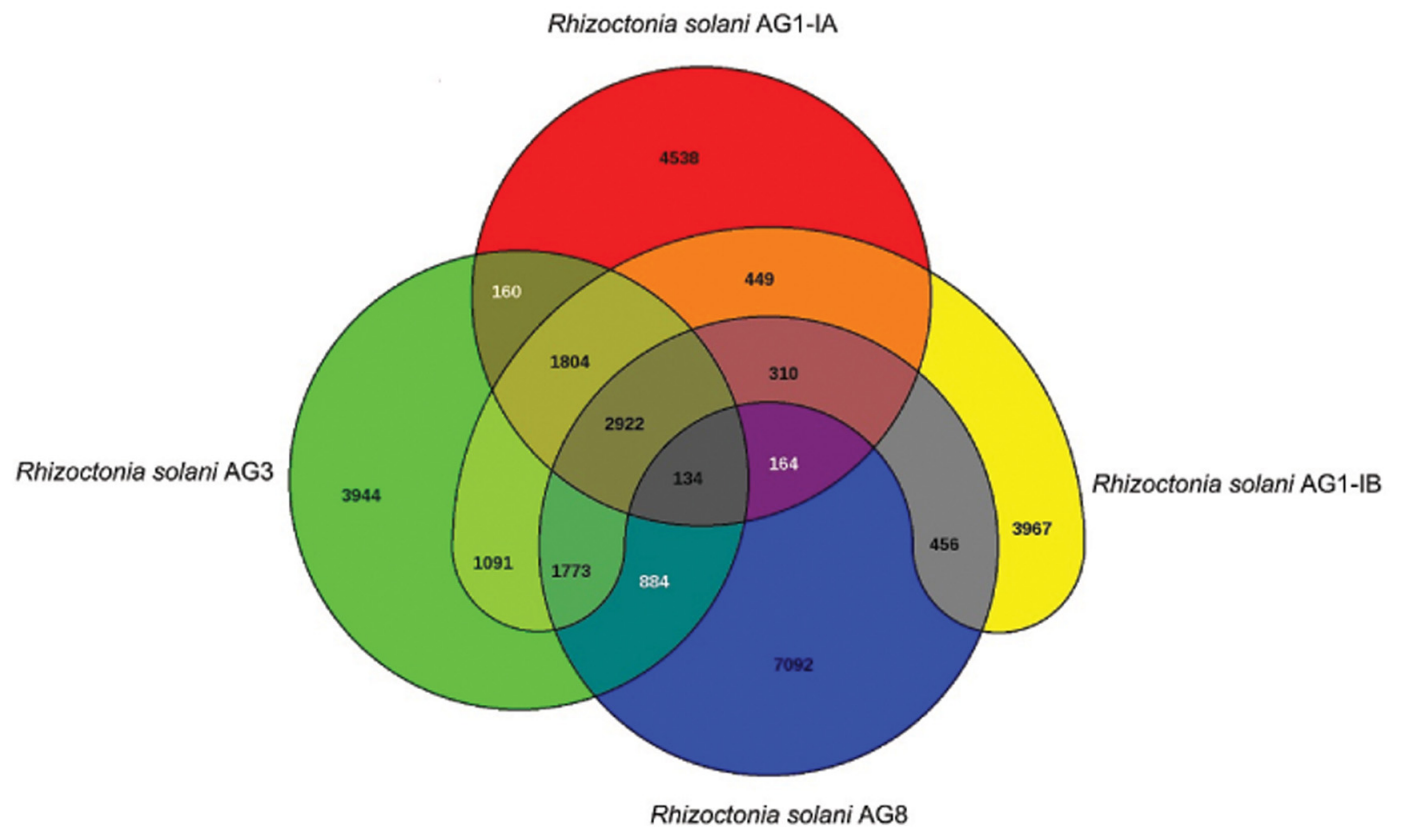
Venn diagram of the gene-based genome comparison for the completely sequenced *R*. *solani* isolates representing AG1-IA, AG1-IB, AG3 and AG8. The core genome of all isolates consists of 2922 genes. These genes are present in the genomes of all sequenced *R*. *solani* isolates: *R*. *solani* AG1-IB 7/3/14 [EMBL:CDGK01000001 –CDGK01018395 (Contigs); LN679100 –LN679996 (Scaffolds)], *R*. *solani* AG3 [GenBank: JATN0100000], *R*. *solani* AG1-IA [Genbank: AFRT00000000] and *R*. *solani* AG8 [GenBank: AVOZ0000000]. For computation of the Venn diagram, default settings of EDGAR [13] were applied.

A gene-based comparative genome analysis for different *R*. *solani* isolates belonging to the different AGs has not been undertaken before because of missing gene information for some isolates. Recently, specific gene predictions for all four *R*. *solani* genomes became available. Gene-based comparisons comprising these annotated genome sequences were performed by means of the comparative genomics tool EDGAR [13] (Fig 3). It appeared that 2922 genes corresponding to 21 to 28% of all genes identified in individual draft genomes represent the core set of genes present in all genomes analysed. Shared genes between sub-sets of *R*. *solani* isolates are depicted in Fig 3. The genomes of the isolates AG1-IB and AG3 possess the largest set of shared genes. In contrast, AG1-IA and AG8 are more distantly related to each other. In first instance, all predicted core genes represent primary house-keeping genes that are expected to be encoded in all *R*. *solani* genomes. In addition, also genes potentially involved in plant cell wall degradation were identified within the core-set of genes (S2 Table).

Comparative analyses also uncovered unique features of each *R*. *solani* isolate (Table 3, S3, S4, S5 and S6 Tables). All isolates possess unique genes potentially involved in the production of plant cell wall degrading enzymes and synthesis of secondary metabolites, e.g. laccase, tannase and cytochromes P450 genes (S7 Table). In comparison to the other isolates, *R*. *solani* AG1-IB 7/3/14 harbours more genes with predictable functions in secondary metabolite synthesis, e.g. non-ribosomal peptide synthesis genes probably involved in siderophore production, terpene synthesis genes and tyrosinase genes having a putative function in melanin-production.

**Table 3:** Examples of predicted unique gene products for *R*. *solani* isolates representing AG1-IA, AG1-IB, AG3 and AG8.

	*R*. *solani* AG1-IA	*R*. *solani* AG1-IB	*R*. *solani* AG3	*R*. *solani* AG8
**Cellulolytic enzymes** ^1^	34	76	95	76
**Tyrosinases** ^1^	2	10	12	9
**Drug resistance proteins** ^1^	19	10	7	5
**Laccases** ^1^	4	8	22	13
**Cytochrome P450** ^1^	46	95	48	38
**Non-ribosomal peptide synthesis enzymes** ^1^	0	2	0	0
**Alkaloid/terpene synthesis enzymes** ^1^	0	2	3	2
**Tannases** ^1^	1	5	4	1
**Volvatoxin-like** ^1^	0	0	1	0

^1^ Corresponding genes were listed in S7 Table

In summary, comparative genome analyses comprising the different completely sequenced *R*. *solani* isolates led to the identification of unique genome features for each isolate. Identified unique genes for different isolates are candidate determinants to explain differences in host range and virulence of corresponding *R*. *solani* isolates.

### Phylogenetic classification of *R*. *solani* isolates based on shared core genes

**Fig 4:**
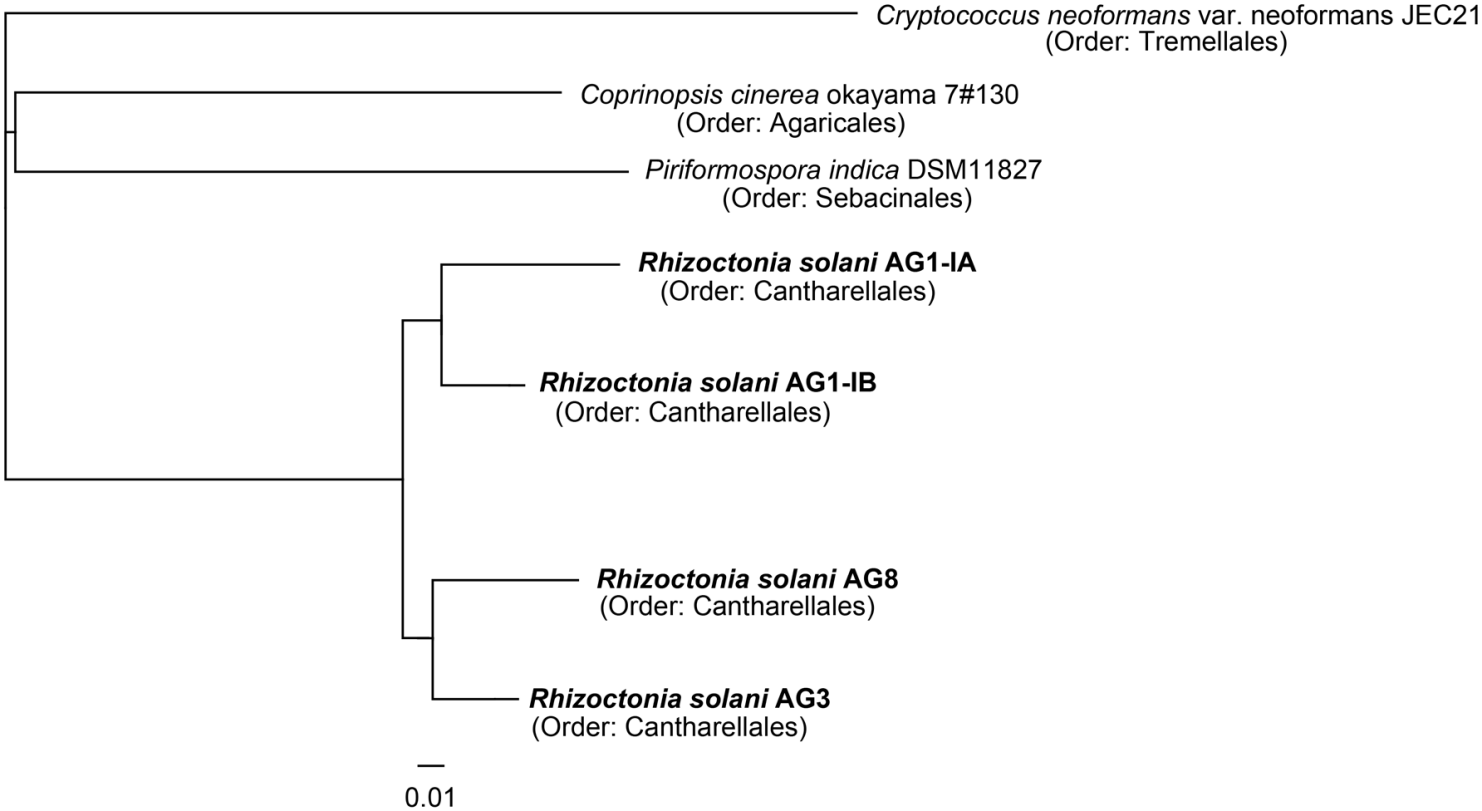
Taxonomic classification of the different *R*. *solani* isolates representing AG1-IA, AG1-IB, AG3 and AG8. The phylogenetic tree is based on all core genes (725) of the selected strains as determined by means of the comparative genomics tool EDGAR [13]. The corresponding tree was calculated within EDGAR. It includes *R*. *solani* AG1-IB (isolate 7/3/14) [3,4], *R*. *solani* AG1-IA (isolate B275) [5], *R*. *solani* AG3 (isolate Rhs1AP) [6], *R*. *solani* AG8 (isolate WAC10335) [7], *C. cinerea okayama 7#130 [24], P. indica DSM 11827 [25] and Cryptococcus neoformans var neoformans JEC21 [26].*

To deduce the phylogeny of the different *R*. *solani* isolates in relation to other completely sequenced members of the phylum *Basidiomycota*, the comparative genomics tool EDGAR was applied. Based on 725 core genes determined for selected species, a phylogenetic tree was computed (Fig 4). The topology of the resulting tree is congruent to the tree calculated for the 18S rRNA marker gene [3]. *R*. *solani* isolates representing the anastomosis groups AG1-IA, AG1-IB, AG3 and AG8 cluster together. *R*. *solani* AG1-IB and *R*. *solani* AG1-IA are more closely related to each other than to *R*. *solani* AG3 and *R*. *solani* AG8. The other fungi of the phylum *Basidiomycota* included in the phylogenetic analysis are only distantly related to *R*. *solani*. *C*. *cinerea* and *P*. *indica* cluster within one group, whereas *C*. *neoformans* only is distantly related to the other fungi. These results are in agreement with previous taxonomic classifications. *C*. *cinerea* and *P*. *indica* belong to the same class (*Agaricomycetes*), whereas *C*. *neoformans* is a member of the class *Tremellomycetes*. To determine similarities within the *R*. *solani* species complex, pairwise Average Nucleotide Identities (ANI) and Average Amino-acid Identities (AAI) were calculated (Table 4 & 5). Usually, genomes of prokaryotic isolates belonging to the same species possess higher ANI and AAI values (above 95%) than those representing different species [28,29].

**Table 4:** Pairwise Average Nucleotide Identity (ANI) analyses for completely sequenced *R*. *solani* isolates.

	AG1-IA	AG1-IB	AG3	AG8
**AG1-IA**	100.00%	81.82%	79.23%	79.09%
**AG1-IB**	-	100.00%	79.29%	79.22%
**AG3**	-	-	100.00%	84.07%
**AG8**	-	-	-	100.00%

**Table 5:** Pairwise Average Amino-acid Identity (AAI) analyses for completely sequenced *R*. *solani* isolates.

	AG1-IA	AG1-IB	AG3	AG8
**AG1-IA**	100.00%	83.74%	79.05%	77.55%
**AG1-IB**	-	100.00%	85.28%	83.22%
**AG3**	-	-	100.00%	88.01%
**AG8**	-	-	-	100.00%

Pairwise comparisons for *R*. *solani* isolates revealed ANI and AAI values below 88% for each pair, indicating considerable sequence differences between isolates representing different anastomosis groups. As a control, ANI and AAI values were also calculated for other fungal strains belonging to the same species (s. Table 6). In these cases, ANI and AAI values above 97% were obtained for isolates representing the same species. However, it should be noted that corresponding genomes were sequenced in their haploid state accounting for a more homogenous genome shape compared to the diploid (heterozygous) state of the *R*. *solani* genomes involving a higher degree of heterogeneity.

**Table 6:** Pairwise Average Amino-acid Identity (AAI) and Average Nucleotide Identity (ANI) analyses for completely sequenced fungal isolates.

Species	Strain 1	Strain 2	ANI	AAI
*Aspergillus niger*	SH2	ATCC1015	98.95%	99.11%
*Candida albicans*	WO-1	A20	98.85%	98.99%
*Crytococcus neoformans* var. neoformans	JEC21	B-3501A	97.89%	98.87%
*Fusarium oxysporum* f.sp. cubense	Race 1	Race 4	97.42%	98.11%
*Metarhizium anisopliae*	BRIP53293	BRIP53284	99.97%	99.98%

However, ANI/AAI values below 88% for pairwise *R*. *solani* comparisons tentatively may suggest that *R*. *solani* isolates representing different anastomosis groups diverged substantially and probably form distinguishable lineages.

## Concluding remarks

Several draft genome sequences for different fungi were established recently by taking advantage of high-throughput sequencing technologies. Application of bioinformatics tools for sequence analysis and interpretation of fungal genomes provided insights into their gene content and life style. However, gene prediction and comparative analyses are still insufficient for this group of microorganisms which mainly is due to the lack of specific gene models for different species and functional gene analyses. Expressed Sequence Tag (EST) and/or high-throughput transcriptome sequencing has only been accomplished for a limited number of species. Hence, application of non-homologous gene models led to inaccuracies in gene predictions. Therefore, important information within fungal genome projects still remains unexplored.

In this work, a new, manually evaluated gene model for an *R*. *solani* AG1-IB isolate was developed and enabled improvements regarding gene prediction and comparative genome analyses among members of the *R*. *solani* species complex. The total number of predicted *R*. *solani* AG1-IB genes may not reflect the complete set of genes for this isolate which mainly is due to the high degree of fragmentation within the obtained draft genome sequence. Genes that are split between contigs cannot be recognized correctly. The multi-cellular and diploid nature of *R*. *solani* AG1-IB 7/3/14 most probably complicates concise genome assemblies and hence detection of the full set of genes within its genome is demanding. Similar problems were reported for other eukaryotic genome projects [36]. Sequencing of the haploid genome status cannot be addressed for *R*. *solani*, since the corresponding phase in its life cycle has not been recognized so far. However, the improved and complemented gene set achieved by application of the *R*. *solani* AG1-IB specific gene model is expected to comprise the great majority of genes encoded in this isolate.

Subsequently to the new gene prediction and annotation of the *R*. *solani* AG1-IB genome, a comparative genome analysis for *R*. *solani* isolates representing different anastomosis groups was enabled. Significant differences between *R*. *solani* genomes were uncovered and now provide the genomic basis for studies addressing host-specificity in pathogenic interactions.

The newly predicted *R*. *solani* AG1-IB 7/3/14 gene set is a valuable resource for evaluation of further RNA-Seq experiments to analyze the pathogenic interaction of the fungus with its host plant. First insights into possible pathogenicity determinants were obtained by *R*. *solani* AG1-IB 7/3/14 high-throughput transcriptome sequencing [8]. However, RNA-Seq analyses for *R*. *solani* AG1-IB in interaction with its host plant lettuce will provide deeper insights into differential transcription of candidate pathogenicity genes. Finally, a better understanding of the function of *R*. *solani* pathogenicity factors is a prerequisite for the development of strategies to control plant diseases caused by this fungus.

## Supporting Information

S1 Table Newly predicted genes in the *R*. *solani* AG1-IB 7/3/14 genome by application of the *R*. *solani* specific gene model. The table provides information on newly predicted genes regarding Gene name, Gene product, Gene function, EC Number, KOG number and KOG functional categories as annotated within the annotation platform GenDBE.
(XLS)

S2 Table *R*. *solani* core genes. The table provides information on KOG and PFAM assignments for all *R*. *solani* core genes.
(XLS)

S3 Table Singleton genes of *R*. *solani* AG1-IA. The table provides information on KOG and PFAM assignments for all *R*. *solani* AG1-IA singleton genes.
(XLS)

S4 Table Singleton genes of *R*. *solani* AG1-IB. The table provides information on KOG and PFAM assignments for all *R*. *solani* AG1-IB singleton genes.
(XLS)

S5 Table Singleton genes of *R*. *solani* AG3. The table provides information on KOG and PFAM assignments for all *R*. *solani* AG3 singleton genes.
(XLS)

S6 Table Singleton genes of *R*. *solani* AG8. The table provides information on KOG and PFAM assignments for all *R*. *solani* AG8 singleton genes.
(XLS)

S7 Table Genes of interest identified in the *R*. *solani* AG1-IA, AG3 and AG8 isolates. The table provides information on KOG and PFAM assignments for genes of interest identified in *R*. *solani* AG1-IA, AG3 and AG8 isolates.
(XLS)
